# Consensus and disparities in perspectives on congenital heart disease management between interventional cardiologists and cardiac surgeons in China

**DOI:** 10.1016/j.xjon.2026.101916

**Published:** 2026-06-10

**Authors:** Xiaofeng Wang, Qinnan Chen, Kai Ma, Benqing Zhang, Hong Gu, Xu Wang

**Affiliations:** a Department of Pediatric Intensive Care Unit, National Center for Cardiovascular Disease and Fuwai Hospital, Chinese Academy of Medical Sciences, Peking Union Medical College, Beijing, China; b Department of Pediatric Cardiac Surgery, National Center for Cardiovascular Disease and Fuwai Hospital, Chinese Academy of Medical Sciences, Peking Union Medical College, Beijing, China; c Department of Pediatric Cardiology, Beijing Anzhen Hospital, Capital Medical University, Beijing, China

**Keywords:** cardiac surgeons, congenital heart disease, interventional cardiologists, questionnaire survey

## Abstract

**Objective:**

This study compares perspectives between interventional cardiologists and cardiac surgeons on congenital heart disease management via a national survey.

**Methods:**

An online questionnaire was distributed to senior surgeons or interventional cardiologists capable of comprehensive congenital heart disease care at 118 hospitals (236 total invitations).

**Results:**

Of 162 valid responses, 90 were surgeons and 72 were cardiologists. There were no differences between surgeons and cardiologists in demographic factors. Survey results showed no statistical differences in (1) the overall relationship between interventional and surgical approaches (complementary and sequential); (2) interventional treatment for complex congenital heart disease (using the Society of Thoracic Surgeons-European Association for Cardio-Thoracic Surgery, simple and complex congenital heart disease were defined as lesions with category I and categories II-V); and (3) multidisciplinary team management (long-term follow-up by a consistent core team). Differences were found in the following areas: 1. In decision-making, surgeons placed emphasis on age and developmental potential (adjusted *P* = .016), whereas cardiologists focused on physiological status and complications (adjusted *P* = .008). 2. For secundum atrial septal defect (*P* < .001), patent ductus arteriosus (*P* < .001), muscular/perimembranous ventricular septal defect (*P* = .001), pulmonary valve stenosis (*P* = .010), and coarctation of the aorta (*P* < .001), each specialty considered their own approach as the preferred option. 3. Surgeons expressed greater demand for interdisciplinary cross-training (adjusted *P* = .020).

**Conclusions:**

Despite broad consensus, cardiologists and surgeons differ in decision-making priorities and training needs. These findings support structured communication, optimized management, specialty-tailored training, and hybrid care models to improve patient outcomes.

**Figure:**
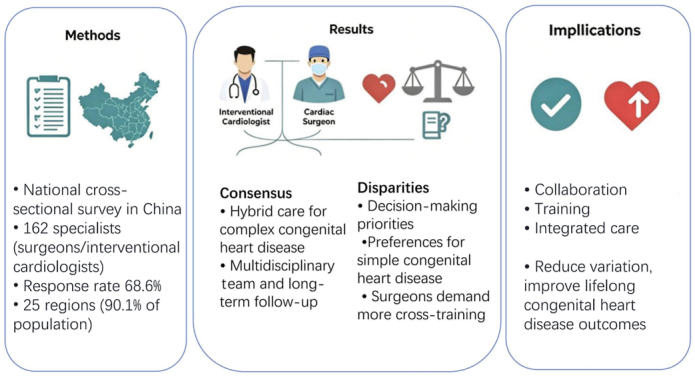
Consensus and disparities in CHD care between Chinese cardiologists and surgeons.

**Central Message**

Specialists agree on hybrid CHD care but differ in decision priorities and training needs. Structured collaboration and tailored training are key to optimizing lifelong patient outcomes.

**Perspective**

This survey highlights that although cardiologists and surgeons agree on the value of hybrid CHD care, they differ in decision-making priorities and training needs. These findings underscore the necessity of structured interdisciplinary communication and specialty-tailored training. Bridging these gaps through optimized MDT models is vital for improving lifelong patient outcomes and advancing CHD care.

Congenital heart disease (CHD) is among the most common congenital malformations worldwide,^1^ with rapidly rising adult survival driven by advances in surgery, perioperative care, and transcatheter interventions.^2^ This has blurred the pediatric–adult care boundary, expanding hybrid procedures that combine surgical and interventional techniques in the same session or planned sequence. This growing population of adults with CHD demands integrated, lifelong care models that bridge pediatric and adult services, driving the need for interdisciplinary hybrid strategies and coordinated transitional care.^3^

Despite these advances, no universal algorithm exists to guide selection between interventional and surgical CHD therapy.^4^ Cepas-Guillén and colleagues^5^ emphasized that lesion anatomy, patient age, institutional volume, and long-term functional consequences must all be carefully weighed during treatment selection. However, they also noted that no standardized or universally accepted decision-making algorithms have been established to guide choices between interventional and surgical therapies. Consequently, treatment decisions depend heavily on local expertise, resource availability, and physicians’ subjective weighting of factors such as procedural invasiveness, reintervention risk, lifelong anticoagulation, and somatic growth potential.

Advances in transcatheter techniques have made hybrid interventions a cornerstone of CHD care, embodying the core collaboration between interventional cardiology and cardiothoracic surgery.^6^ Hybrid approaches overcome limitations of size, vascular access, illness severity, and anatomy that challenge surgery or percutaneous techniques alone. However, key questions remain: how these 2 specialties prioritize clinical factors for the same patient, how to facilitate interdisciplinary collaboration and multidisciplinary team (MDT) consultations, and what training these physicians require.

We therefore conducted a nationwide survey to quantify consensus and divergence in CHD management, identify areas where differing priorities may lead to inconsistent care, and build an evidence base for joint training and interdisciplinary decision-making tools to support lifelong hybrid CHD care in China and beyond.

## Materials and Methods

### Study Design and Participants

A multicenter, cross-sectional survey was conducted among CHD specialists. The methodology, based on existing literature, clinical guidelines, and preliminary frameworks, ensured scientific rigor and clinical relevance.

Inclusion criteria included work in tertiary hospitals (general hospitals, or children's hospitals) with established CHD diagnosis and treatment capabilities (a total of 118 hospitals^7^) and for each participating hospital, 1 interventional cardiologist and 1 cardiac surgeon were invited to complete the survey. Eligible clinicians were department heads or had at least 10 years of independent clinical decision-making experience in CHD care. A total of 236 questionnaires were distributed.

This sampling strategy reflects the clinical governance structure of CHD care in Chinese tertiary hospitals, where senior specialists and department heads hold primary authority for treatment decisions, including the choice between surgery and interventional catheterization procedures. Their experience and leadership make them representative of departmental practice patterns. Additionally, in our country, cardiologists and cardiac surgeons work in separate departments. Accordingly, they maintain independence in both clinical work and questionnaire responses.

Surveys were excluded from analysis if they met any of the following conditions: no response was received, questionnaire responses were incomplete, or duplicate responses were identified by IP address and professional data. This study was approved by our institutional Ethics Committee (ID: 2025-2833; approval date: September 4, 2025). A waiver of written informed consent was granted due to the anonymous online survey design. All participants were informed of the study purpose, data use, and anonymity guarantees at the start of the questionnaire, and submission of the survey constituted implied informed consent.

### Questionnaire Development

The survey items were designed based on published surveys, guideline controversies, and real-world practice dilemmas in daily CHD care, covering 6 core domains: demographics, the relationship between interventional and surgical approaches, management of simple/complex CHD (using the Society of Thoracic Surgeons–European Association for Cardio-Thoracic Surgery risk classification, simple CHD was defined as category I lesions and complex CHD as categories II-V lesions), clinical decision factors, MDT collaboration, and training needs.

Construct validity was established through theoretical domain division. All items were logically assigned to the 6 predefined dimensions according to professional meaning and study aims, with consistent response patterns within domains and clear differences across domains, confirming satisfactory construct validity.

The initial questionnaire was reviewed by 5 experts to evaluate content validity. Items were rated on a 4-point relevance scale. The item-level content validity index was 0.80 of more for all items, and the scale-level index was 0.92, indicating excellent content validity. A pre-survey among 10 physicians refined unclear items. The final questionnaire was distributed, and a Cronbach's α 0.70 or greater confirmed acceptable internal consistency. The translated version of the original questionnaire used in this study is presented as a Appendix E1.

### Data Collection

The electronic questionnaire was distributed online from November to December 2025. Participants received detailed instructions on the study purpose and completion. After collection, all retrieved questionnaires were rigorously screened per predefined exclusion criteria. Valid questionnaires were compared by specialty to analyze differences in CHD management.

### Statistical Analysis

Statistical analysis was performed using SPSS 26.0 (IBM). Categorical variables were presented as numbers, percentages, and 95% CIs. Continuous variables (years of independent decision-making, annual case volume) were dichotomized using clinical thresholds: 10 to 19 years versus 20 years or more, and less than 200 versus 200 or more cases/year.

For 2 × 2 tables, Pearson's chi-square test was used if all expected counts 5 or more, continuity-corrected chi-square was used for counts 1 to 4, and the Fisher exact test was used if any count less than 1 or more than 20% of counts less than 5. For 2 × R tables, Pearson's chi-square test was applied if 80% or more of expected counts 5 or more and no count less than 1; otherwise, the Fisher exact test was used.

For multi-category items, global *P* values were reported. Significant global tests were followed by Bonferroni-corrected post hoc pairwise comparisons, with adjusted *P* values presented to control type I error.

## Results

After excluding 74 nonresponses, 162 valid questionnaires were included, with an overall response rate of 68.6%. Surgeons accounted for 90 of 118 (76.3%) and cardiologists accounted for 72 of 118 (61.0%). Respondents were from 25 provincial-level regions in China, covering approximately 1.269 billion people, or 90.1% of the national population (2024 census).

Demographic characteristics were well balanced between the 2 groups (Table 1), with no significant differences observed in hospital type, teaching status, hospital ownership, regional economic level, years of independent clinical decision-making experience, age-spectrum coverage, or annual individual procedural volume.

**Table 1:** Demographic characteristics of questionnaire respondents

Items	Surgeon N = 90	Cardiologist N = 72	*P* value
Nature of Hospital			.876
General Hospital	N = 64 (71.1%, 95% CI, 61.0-79.5%)	N = 52 (72.2%, 95% CI, 61.0-81.2%)	
Children's Hospital	N = 26 (28.9%, 95% CI, 20.5-39.0%)	N = 20 (27.8%, 95% CI, 18.8-39.0%)	
Teaching status			.920
Teaching Hospital	N = 77 (85.6%, 95% CI, 76.8-91.4%)	N = 62 (86.1%, 95% CI, 76.3-92.3%)	
Nonteaching Hospital	N = 13 (14.4%, 95% CI, 8.6-23.2%)	N = 10 (13.9%, 95% CI, 7.7-23.7%)	
Hospital ownership			.133
Public Hospital	N = 81 (90.0%, 95% CI, 82.3-94.8%)	N = 70 (97.2%, 95% CI, 94.8-100%)	
Private Hospital	N = 9 (10.0%, 95% CI, 5.2-17.7%)	N = 2 (2.8%, 95% CI, 0.4-9.0%)	
Gross domestic product per capita at the provincial level			.885
Above the national level	N = 56 (62.2%, 95% CI, 52.2-72.2%)	N = 44 (61.1%, 95% CI, 49.8-72.4%)	
Below the national level	N = 34 (37.8%, 95% CI, 27.8-47.8%)	N = 28 (38.9%, 95% CI, 27.6-50.2%)	
Years of independent decision-making			.502
10-19 y	N = 62 (68.9%, 95% CI, 58.7-77.5%)	N = 46 (63.9%, 95% CI, 52.4-74.0%)	
≥20 y	N = 28 (31.1%, 95% CI, 22.5-41.3%)	N = 26 (36.1%, 95% CI, 26.0-47.6%)	
Patients cover all age groups			.672
Yes	N = 43 (47.8%, 95% CI, 37.8-58.0%)	N = 32 (44.4%, 95% CI, 33.5-55.9%)	
No	N = 47 (52.2%, 95% CI, 42.0-62.2%)	N = 40 (55.6%, 95% CI, 44.1-66.5%)	
Individual surgery/intervention volume			.450
≥200 cases/y	N = 26 (28.9%, 95% CI, 20.5-39.0%)	N = 17 (23.6%, 95% CI, 14.9-35.3%)	
<200 cases/y	N = 64 (71.1%, 95% CI, 61.0-79.5%)	N = 55 (76.4%, 95% CI, 64.7-85.1%)	

Surgeons and cardiologists showed high consensus in their overall perception of interventional and surgical treatments (Table 2), with most respondents viewing the 2 approaches as complementary and sequential.

**Table 2:** The overall perception of interventional and surgical treatments

Items	Surgeon N = 90	Cardiologist N = 72	*P* value
Positioning of interventional and surgical treatment across the entire disease course			.061
A: Complementary and sequential relationship: Interventional and surgical approaches serve as the preferred options for different stages and types of lesions, jointly forming a complete treatment pathway	N = 48 (53.3%, 95% CI, 43.0-63.4%)	N = 46 (63.9%, 95% CI, 52.4-74.0%)	
B: Competition and substitution relationship: With technological advances, interventional therapy is progressively replacing surgery as the first-line treatment for an increasing number of lesions	N = 0 (0.0%, 95% CI, 0.0-4.1%)	N = 2 (2.8%, 95% CI, 0.8-9.6%)	
C: Integration and hybridization relationship: The future direction lies in the deep convergence of interventional and surgical disciplines, combining both techniques within a single procedure rather than engaging in simple comparisons	N = 26 (28.9%, 95% CI, 20.5-39.0%)	N = 10 (13.9%, 95% CI, 7.7-23.7%)	
D: Stratification and selection relationship: The decision-making hinges on a refined assessment of anatomy, physiology, patient age, and institutional expertise; there is no universally applicable preferred approach	N = 16 (17.8%, 95% CI, 11.1-27.0%)	N = 14 (19.4%, 95% CI, 11.7-30.3%)	

Significant intergroup differences were identified in clinical decision-making priorities (Table 3). Surgeons prioritized age and growth potential (adjusted *P* = .016), whereas cardiologists focused on physiological status and complications (adjusted *P* = .008).

**Table 3:** The decision-making process (global *P* = .013)

Items	Surgeon N = 90	Cardiologist N = 72	Adjusted *P* value
Apart from the lesion type, which of the following factors do you think has the greatest impact?			
A: Age and growth potential: Children require consideration of future growth and secondary interventions, while adults focus more on immediate outcomes and durability.	N = 60 (66.7%, 95% CI, 56.3-75.6%)	N = 32 (44.4%, 95% CI, 33.5-55.9%)	.016
B: Physiological status and complications: urgent physiological conditions in neonates/infants, including cardiogenic shock with metabolic acidosis, severe refractory hypoxemia and intractable heart failure; complications such as arrhythmias, pulmonary hypertension, and heart failure in adults.	N = 16 (17.8%, 95% CI, 11.1-27.0%)	N = 28 (38.9%, 95% CI, 28.3-50.6%)	.008
C: Previous surgical history and anatomical changes: Adhesions and anatomic distortions resulting from multiple surgeries significantly affect the choice of subsequent strategies.	N = 4 (4.4%, 95% CI, 1.7-10.8%)	N = 6 (8.3%, 95% CI, 3.9-17.1%)	>.999
D: Patient/family preferences and psychosocial factors: Considerations regarding trauma, scarring, recovery time, treatment costs, and insurance coverage vary significantly across different age groups.	N = 10 (11.1%, 95% CI, 6.1-19.3%)	N = 6 (8.3%, 95% CI, 3.9-17.1%)	>.999

For simple CHD, including secundum atrial septal defect (*P* < .001), patent ductus arteriosus (*P* < .001), muscular/perimembranous ventricular septal defect (*P* = .001), pulmonary valve stenosis (*P* = .010), and coarctation of the aorta (*P* < .001), there was a significant divergence between cardiologists and surgeons: Cardiologists strongly favored interventional therapy as first-line management, whereas surgeons preferred surgical approaches for these simple CHD lesions (Table 4). Both groups strongly supported MDT care, with no significant difference in their ranking of core MDT characteristics (Table 5). Regarding professional training needs (Table 6), surgeons expressed a significantly higher demand for interdisciplinary cross-training (adjusted *P* = .020).

**Table 4:** Interventional technology in specific lesions: Maturity and status

Items	Surgeon N = 90	Cardiologist N = 72	*P* value
Secundum atrial septal defect			<.001
A: Remains at an investigative stage with a narrow scope of application.	N = 8 (8.9%, 95% CI, 4.5-16.8%)	N = 0 (0.0%, 95% CI, 0.0-5.1%)	
B: It has become an important option, but not the first-line choice.	N = 30 (33.3%, 95% CI, 24.1-44.0%)	N = 6 (8.3%, 95% CI, 3.9-16.9%)	
C: It is now the preferred/first-line therapy for specific lesions.	N = 52 (57.8%, 95% CI, 47.3-67.7%)	N = 66 (91.7%, 95% CI, 83.1-96.1%)	
Patent ductus arteriosus			<.001
A: Remains at an investigative stage with a narrow scope of application.	N = 4 (4.4%, 95% CI, 1.7-10.8%)	N = 0 (0.0%, 95% CI, 0.0-5.1%)	
B: It has become an important option, but not the first-line choice.	N = 30 (33.3%, 95% CI, 24.1-44.0%)	N = 6 (8.3%, 95% CI, 3.9--16.9%)	
C: It is now the preferred/first-line therapy for specific lesions.	N = 56 (62.2%, 95% CI, 51.7-71.7%)	N = 66 (91.7%, 95% CI, 83.1-96.1%)	
Muscular/perimembranous ventricular septal defect			.001
A: Remains at an investigative stage with a narrow scope of application.	N = 38 (42.2%, 95% CI, 32.4-52.7%)	N = 12 (16.7%, 95% CI, 9.8-27.1%)	
B: It has become an important option, but not the first-line choice.	N = 30 (33.3%, 95% CI, 24.1-44.0%)	N = 30 (41.7%, 95% CI, 30.8-53.4%)	
C: It is now the preferred/first-line therapy for specific lesions.	N = 22 (24.4%, 95% CI, 16.4-34.6%)	N = 30 (41.7%, 95% CI, 30.8-53.4%)	
Balloon angioplasty for pulmonary valve stenosis			.010
A: Remains at an investigative stage with a narrow scope of application.	N = 10 (11.1%, 95% CI, 6.1-19.3%)	N = 4 (5.6%, 95% CI, 2.2-13.5%)	
B: It has become an important option, but not the first-line choice.	N = 42 (46.7%, 95% CI, 36.5-57.1%)	N = 20 (27.8%, 95% CI, 18.6-39.5%)	
C: It is now the preferred/first-line therapy for specific lesions.	N = 38 (42.2%, 95% CI, 32.4-52.7%)	N = 48 (66.7%, 95% CI, 55.2-76.7%)	
Transcatheter pulmonary valve replacement			.116
A: Remains at an investigative stage with a narrow scope of application.	N = 46 (51.1%, 95% CI, 40.8-61.3%)	N = 28 (38.9%, 95% CI, 28.3-50.6%)	
B: It has become an important option, but not the first-line choice.	N = 34 (37.8%, 95% CI, 28.3-48.4%)	N = 28 (38.9%, 95% CI, 28.3-50.6%)	
C: It is now the preferred/first-line therapy for specific lesions.	N = 10 (11.1%, 95% CI, 6.1-19.3%)	N = 16 (22.2%, 95% CI, 13.9-33.4%)	
Stent implantation for pulmonary artery stenosis			.055
A: Remains at an investigative stage with a narrow scope of application.	N = 38 (42.2%, 95% CI, 32.4-52.7%)	N = 18 (25.0%, 95% CI, 16.1-36.9%)	
B: It has become an important option, but not the first-line choice.	N = 38 (42.2%, 95% CI, 32.4-52.7%)	N = 36 (50.0%, 95% CI, 38.7-61.3%)	
C: It is now the preferred/first-line therapy for specific lesions.	N = 14 (15.6%, 95% CI, 9.5-24.6%)	N = 18 (25.0%, 95% CI, 16.1-36.9%)	
Coarctation of the aorta (patient age 1-10/12 y: balloon angioplasty; ≥10-12 y: stent implantation)			<.001
A: Remains at an investigative stage with a narrow scope of application.	N = 34 (37.8%, 95% CI, 28.3-48.4%)	N = 14 (19.4%, 95% CI, 11.7-30.3%)	
B: It has become an important option, but not the first-line choice.	N = 52 (57.8%, 95% CI, 47.3-67.7%)	N = 36 (50.0%, 95% CI, 38.7-61.3%)	
C: It is now the preferred/first-line therapy for specific lesions.	N = 4 (4.4%, 95% CI, 1.7-10.8%)	N = 22 (30.6%, 95% CI, 20.9-42.3%)	
Various fistulae (eg, coronary artery fistula, pulmonary arteriovenous fistula)			.393
A: Remains at an investigative stage with a narrow scope of application.	N = 32 (35.6%, 95% CI, 26.3-46.0%)	N = 20 (27.8%, 95% CI, 18.6-39.5%)	
B: It has become an important option, but not the first-line choice.	N = 36 (40.0%, 95% CI, 30.2-50.5%)	N = 28 (38.9%, 95% CI, 28.3-50.6%)	
C: It is now the preferred/first-line therapy for specific lesions.	N = 22 (24.4%, 95% CI, 16.4-34.6%)	N = 24 (33.3%, 95% CI, 23.3-45.0%)	
Fontan-associated complications (eg, conduit stenosis, lymphatic circulation disorders, fenestration closure)			.9
A: Remains at an investigative stage with a narrow scope of application.	N = 28 (31.1%, 95% CI, 22.5-41.3%)	N = 22 (30.6%, 95% CI, 20.9-42.3%)	
B: It has become an important option, but not the first-line choice.	N = 22 (24.4%, 95% CI, 16.4-34.6%)	N = 20 (27.8%, 95% CI, 18.6-39.5%)	
C: It is now the preferred/first-line therapy for specific lesions.	N = 40 (44.4%, 95% CI, 34.5-54.8%)	N = 30 (41.7%, 95% CI, 30.8-53.4%)	
d-Transposition of the great arteries with intact ventricular septum			.081
A: Remains at an investigative stage with a narrow scope of application.	N = 50 (55.6%, 95% CI, 45.1-65.6%)	N = 30 (41.7%, 95% CI, 30.8-53.4%)	
B: It has become an important option, but not the first-line choice.	N = 32 (35.6%, 95% CI, 26.3-46.0%)	N = 28 (38.9%, 95% CI, 28.3-50.6%)	
C: It is now the preferred/first-line therapy for specific lesions	N = 8 (8.9%, 95% CI, 4.5-16.8%)	N = 14 (19.4%, 95% CI, 11.7-30.3%)	
Transcatheter occlusion of major aortopulmonary collateral arteries			.41
A: Remains at an investigative stage with a narrow scope of application.	N = 16 (17.8%, 95% CI, 11.1-27.0%)	N = 8 (11.1%, 95% CI, 5.7-20.5%)	
B: It has become an important option, but not the first-line choice.	N = 26 (28.9%, 95% CI, 20.5-39.0%)	N = 26 (36.1%, 95% CI, 25.8-47.8%)	
C: It is now the preferred/first-line therapy for specific lesions.	N = 48 (53.3%, 95% CI, 43.0-63.4%)	N = 38 (52.8%, 95% CI, 41.4-63.9%)

**Table 5:** Multidisciplinary team

Items	Surgeon N = 90	Cardiologist N = 72	*P* value
The most important characteristic of the MDT in managing patients across the entire life course			.215
A: True physical integration: the availability of a hybrid operating room to facilitate seamless collaboration between surgical and interventional teams.	N = 12 (13.3%, 95% CI, 7.7-22.1%)	N = 4 (5.6%, 95% CI, 2.2-13.5%)	
B: Institutionalized joint decision-making process: weekly, fixed-schedule MDT meetings with mandatory discussion of all complex cases.	N = 32 (35.6%, 95% CI, 26.3-46.0%)	N = 20 (27.8%, 95% CI, 18.6-39.5%)	
C: Shared patient data platform: an electronic medical record system integrating imaging, cardiac catheterization, and surgical records.	N = 4 (4.4%, 95% CI, 1.7-10.8%)	N = 2 (2.8%, 95% CI, 0.8-9.6%)	
D: Longitudinal care by the same core team: long-term follow-up by a core physician team familiar with the patient's “complete medical history.”	N = 38 (42.2%, 95% CI, 32.4-52.7%)	N = 40 (55.6%, 95% CI, 44.1-66.5%)	
E: A robust transition care system: a dedicated team ensures the seamless transfer of adolescents from pediatric to adult healthcare.	N = 4 (4.4%, 95% CI, 1.7-10.8%)	N = 6 (8.3%, 95% CI, 3.9-16.9%)	

*MDT*, Multidisciplinary team.

**Table 6:** Training of congenital heart disease specialists (global *P* = .002)

Items	Surgeon N = 90	Cardiologist N = 72	Adjusted *P* value
In your opinion, what is the most pressing need for improvement in the current system?			
A: Creation of a structured training and accreditation pathway for an independent subspecialty in CHD treatment.	N = 8 (8.9%, 95% CI, 4.5-16.8%)	N = 16 (22.2%, 95% CI, 13.9-33.4%)	.085
B: Enhance cross-training between interventional and surgical disciplines, for instance, by having interventionalists learn the surgical perspective and surgeons master basic catheter skills.	N = 42 (46.7%, 95% CI, 36.5-57.1%)	N = 18 (25.0%, 95% CI, 16.1-36.9%)	.020
C: Establish comprehensive training programs that cover both pediatric and adult CHD, thereby bridging the patient-age divide.	N = 36 (40.0%, 95% CI, 30.2-50.5%)	N = 32 (44.4%, 95% CI, 33.5-55.9%)	>.999
D: Promote the inclusion of interventional cardiologists as core members of the surgical team during complex CHD procedures.	N = 0 (0.0%, 95% CI, 0.0-4.1%)	N = 4 (5.6%, 95% CI, 2.2-13.5%)	.145
E: Increase access to simulation-based training targeting rare and high-risk procedures.	N = 4 (4.4%, 95% CI, 1.7-10.8%)	N = 2 (2.8%, 95% CI, 0.8-9.6%)	>.999

*CHD*, Congenital heart disease.

## Discussion

This national survey of interventional cardiologists and cardiac surgeons from 118 Chinese hospitals identified key consensus and disparities in CHD management. Although both specialties agreed on therapeutic principles, interventional roles in complex CHD, and lifelong MDT care, they differed notably in clinical decision priorities, treatment preferences for simple CHD, and training needs. These findings provide empirical evidence to optimize collaborative care and professional training for CHD, which outlines the study design, key inter-specialty consensus and disparities, and clinical implications for standardized lifelong CHD care delivery.

More than half of respondents viewed interventional and surgical therapies as complementary and sequential, consistent with the global trend of hybrid CHD management.^8^ Preinterventional optimization can lower surgical risk for complex CHD, and postoperative intervention may resolve residual lesions.^9^ Both specialties acknowledged the value of interventional techniques, whose indispensable role underpins multidisciplinary collaboration in complex CHD care.

Consistent with our results, international studies have also demonstrated the growing value and safety of interventional therapy for CHD. Linnane and colleagues^10^ reviewed risk stratification and novel low-risk percutaneous strategies in CHD, confirming their efficacy in reducing adverse outcomes and advocating technical innovation beyond mortality end points. Brida and colleagues^11^ reported favorable safety profiles in a large cohort of adult patients with CHD undergoing catheter interventions, noting a trend toward increasingly complex procedures while maintaining low complication rates.

For simple CHD, physicians tend to favor their own specialty's interventions. Although this disciplinary preference reflects routine clinical practice, it may introduce treatment bias. Recent atrial septal defect studies indicate interventional occlusion and surgical repair are not mutually exclusive but should be tailored to patient age, defect anatomy, and comorbidities.^12^ Our observed preference differences underscore the need for standardized selection criteria and MDT-driven decision-making integrating anatomic and individual patient factors to avoid 1-sided disciplinary bias.

Notably, only 9.9% of physicians in this study addressed insurance-coverage issues, despite adults with CHD facing substantial insurance and financial challenges.^13^ This disconnect may reflect China's healthcare system: Universal basic medical insurance provides fundamental financial protection, whereas social workers and public welfare foundations offer additional support, partially mitigating financial barriers. Although existing policies have largely eased patients' current financial strain, advances in medical techniques, devices, and pharmaceuticals create new clinical needs. Thus, medical insurance and public welfare funds must evolve accordingly. Clinicians should proactively screen for patients facing financial hardship, coordinate insurance benefits and social assistance, and drive timely updates to supportive programs. Since CHD requires lifelong continuous care, failure to address these evolving financial needs will undermine patients' long-term treatment adherence. For this reason, this gap still warrants urgent attention from clinicians involved in lifelong CHD care.

Both groups regarded long-term follow-up by a core team familiar with patients’ full medical histories as the core component of MDT, which aligns well with the lifelong management needs of patients with CHD. A Japanese adult CHD referral study identified insufficient continuous MDT follow-up as a major cause of treatment interruption and increased complications.^14^ Patients with complex CHD require long-term monitoring of cardiac function, vascular remodeling, and associated complications, needs that cannot be adequately addressed by a single discipline.^15^

The study's consensus confirms that the core value of MDT lies in ensuring lifelong continuity of care and personalized management, which is critical for addressing long-term sequelae (eg, pulmonary hypertension, arrhythmias) in adult patients with CHD. These findings highlight an urgent need to formalize transitional and lifelong care competencies to avoid management gaps,^16^ providing a clear rationale for establishing MDT models in national CHD centers.

Surgeons prioritize age and developmental potential, whereas cardiologists focus on age, physiological status, and comorbidities, a divergence inherent to their respective practices. Surgery is limited by weight, thoracic development, and surgical tolerance, especially in complex pediatric CHD where growth impacts outcomes. Interventional therapy, by contrast, suits patients with comorbidities and poor surgical tolerance, requiring comprehensive physiological and risk assessment. These differing priorities highlight the need for interdisciplinary collaboration integrating anatomic, physiological, and patient-related factors to enable balanced, patient-centered multidisciplinary CHD care.^17^

Surgeons and cardiologists differed in their training needs for CHD care. After Bonferroni adjustment, surgeons showed significantly higher demand for interdisciplinary cross-training, placing greater emphasis on interventional skill learning. This discrepancy reflects disciplinary development and inherent limitations. With interventional therapy increasingly applied to simple CHD, surgeons need basic interventional competencies to broaden their diagnostic and therapeutic scope. Such training is reasonable and has been shown to enhance surgeons’ capability in managing complex CHD cases.^18^

### Limitations

This study has several limitations. First, it relies on self-reported views of surgeons and interventional cardiologists, which may not fully reflect real clinical decisions, and excludes noninterventional cardiologists who contribute to multidisciplinary care. Second, although participants were recruited across 25 provinces, primary hospital physicians were not included, restricting generalizability to all CHD practitioners. Third, only senior decision-making specialists were surveyed, omitting junior clinicians’ perspectives on hybrid procedures. Fourth, all participants were Chinese tertiary hospital specialists, limiting global applicability. Last, the impact of observed consensus and disparities on patient outcomes were not evaluated. Future work should develop and validate interdisciplinary training and standardized MDT pathways, assessing their effects on clinical practice and long-term cardiac outcomes.

## Conclusions

On the basis of the results of our study, we propose actionable strategies to mitigate practice variations and advance patient-centered care for individuals with CHD. Recommended measures include regular MDT case conferences for complex lesions, standardized clinical pathways for common simple defects, enhanced interdisciplinary cross-training, unified patient-centered decision-making frameworks that integrate anatomic and physiological considerations, and routine evaluation of financial barriers supported by medical insurance and social welfare resources. These approaches prioritize optimized care coordination over rigid professional uniformity, while acknowledging inherent disciplinary differences that align with global clinical trends. Collectively, they streamline clinical decision-making, improve care consistency, and facilitate individualized, equitable, and long-term outcome-oriented management for patients with CHD.

## Conflict of Interest Statement

The authors reported no conflicts of interest.

The *Journal* policy requires editors and reviewers to disclose conflicts of interest and to decline handling or reviewing manuscripts for which they may have a conflict of interest. The editors and reviewers of this article have no conflicts of interest.

## Appendix E1 Survey on perspectives in congenital heart disease management—A multidisciplinary view from interventional cardiology and cardiac surgery

Dear Expert,

Greetings!

We are the pediatric intensive care unit team from Fuwai Hospital. With years of frontline clinical experience in the perioperative management and long-term follow-up of patients with congenital heart disease (CHD), we deeply understand that optimal CHD management requires close multidisciplinary collaboration, particularly between interventional cardiologists and cardiac surgeons. Furthermore, the safety and effectiveness of perioperative care are highly dependent on sound decision-making in the upstream treatment strategies.

To accurately understand the consensus and differences in perspectives among physicians from different specialties regarding CHD management, optimize multidisciplinary collaboration models, and ultimately align perioperative care more closely with clinical treatment needs, we have designed this questionnaire. The survey primarily targets interventional cardiologists, cardiac surgeons, and all relevant professionals involved in CHD diagnosis and treatment.

The questionnaire is anonymous and takes approximately 5 to 10 minutes to complete, with single-choice answers for each question. All data collected will be used solely for academic research purposes.

We sincerely appreciate your support!

1. __Your Profession

A: Surgeon

B: Cardiologist

2. __Nature of Hospital

A: General Hospital

B: Children's Hospital

3. __ Teaching Status

A: Teaching Hospital

B: Nonteaching Hospital

4. __ Hospital Ownership

A: Public Hospital

B: Private Hospital

5. __Years of Independent Decision-making

A: 10-19 years

B: ≥20 years

6. __ Patients Cover All Age Groups

A: Yes

B: No

7. __ Individual Surgery/Intervention Volume

A: ≥200 cases/year

B: <200 cases/year

8. __ Gross Domestic Product per Capita at the Provincial Level

A: Above the national level

B: Below the national level

9. __ The overall perception of interventional and surgical treatments: Positioning of interventional and surgical treatment across the entire disease course.

A: Complementary and sequential relationship: Interventional and surgical approaches serve as the preferred options for different stages and types of lesions, jointly forming a complete treatment pathway.

B: Competition and substitution relationship: With technological advances, interventional therapy is progressively replacing surgery as the first-line treatment for an increasing number of lesions.

C: Integration and hybridization relationship: The future direction lies in the deep convergence of interventional and surgical disciplines, combining both techniques within a single procedure rather than engaging in simple comparisons.

D: Stratification and selection relationship: The decision-making hinges on a refined assessment of anatomy, physiology, patient age, and institutional expertise; there is no universally applicable preferred approach.

10. __ The decision-making process: Apart from the lesion type, which of the following factors do you think has the greatest impact?

A: Age and Growth Potential: Children require consideration of future growth and secondary interventions, whereas adults focus more on immediate outcomes and durability.

B: Physiological Status and Complications: Urgent physiological conditions in neonates/infants, including cardiogenic shock with metabolic acidosis, severe refractory hypoxemia and intractable heart failure; complications such as arrhythmias, pulmonary hypertension, and heart failure in adults.

C: Previous Surgical History and Anatomical Changes: Adhesions and anatomical distortions resulting from multiple surgeries significantly affect the choice of subsequent strategies.

D: Patient/Family Preferences and Psychosocial Factors: Considerations regarding trauma, scarring, recovery time, treatment costs, and insurance coverage vary significantly across different age groups.

11. __ Interventional Technology in Specific Lesions: Secundum Atrial Septal Defect

A: Remains at an investigative stage with a narrow scope of application.

B: It has become an important option, but not the first-line choice.

C: It is now the preferred/first-line therapy for specific lesions.

12. __ Interventional Technology in Specific Lesions: Patent Ductus Arteriosus

A: Remains at an investigative stage with a narrow scope of application.

B: It has become an important option, but not the first-line choice.

C: It is now the preferred/first-line therapy for specific lesions.

13. __ Interventional Technology in Specific Lesions: Muscular/Perimembranous Ventricular Septal Defect

A: Remains at an investigative stage with a narrow scope of application.

B: It has become an important option, but not the first-line choice.

C: It is now the preferred/first-line therapy for specific lesions.

14. __ Interventional Technology in Specific Lesions: Balloon Angioplasty for Pulmonary Valve Stenosis

A: Remains at an investigative stage with a narrow scope of application.

B: It has become an important option, but not the first-line choice.

C: It is now the preferred/first-line therapy for specific lesions.

15. __ Interventional Technology In Specific Lesions: Transcatheter Pulmonary Valve Replacement

A: Remains at an investigative stage with a narrow scope of application.

B: It has become an important option, but not the first-line choice.

C: It is now the preferred/first-line therapy for specific lesions.

16. __ Interventional Technology in Specific Lesions: Stent Implantation for Pulmonary Artery Stenosis

A: Remains at an investigative stage with a narrow scope of application.

B: It has become an important option, but not the first-line choice.

C: It is now the preferred/first-line therapy for specific lesions.

17. __ Interventional Technology in Specific Lesions: Coarctation of the Aorta (patient age 1-10/12 years: balloon angioplasty; ≥10-12 years: stent implantation)

A: Remains at an investigative stage with a narrow scope of application.

B: It has become an important option, but not the first-line choice.

C: It is now the preferred/first-line therapy for specific lesions.

18. __ Interventional Technology in Specific Lesions: Various Fistulae (eg, coronary artery fistula, pulmonary arteriovenous fistula)

A: Remains at an investigative stage with a narrow scope of application.

B: It has become an important option, but not the first-line choice.

C: It is now the preferred/first-line therapy for specific lesions.

19. __ Interventional Technology in Specific Lesions: Fontan-associated Complications (eg, conduit stenosis, lymphatic circulation disorders, fenestration closure)

A: Remains at an investigative stage with a narrow scope of application.

B: It has become an important option, but not the first-line choice.

C: It is now the preferred/first-line therapy for specific lesions.

20. __ Interventional Technology in Specific Lesions: d-Transposition of the Great Arteries With Intact Ventricular Septum

A: Remains at an investigative stage with a narrow scope of application.

B: It has become an important option, but not the first-line choice.

C: It is now the preferred/first-line therapy for specific lesions.

21. __ Interventional Technology in Specific Lesions: Transcatheter Occlusion of Major Aortopulmonary Collateral Arteries

A: Remains at an investigative stage with a narrow scope of application.

B: It has become an important option, but not the first-line choice.

C: It is now the preferred/first-line therapy for specific lesions.

22. __ Multidisciplinary Team (MDT): The Most Important Characteristic of the MDT in Managing Patients Across the Entire Life Course

A: True physical integration: the availability of a hybrid operating room to facilitate seamless collaboration between surgical and interventional teams.

B: Institutionalized joint decision-making process: weekly, fixed-schedule MDT meetings with mandatory discussion of all complex cases.

C: Shared Patient Data Platform: An Electronic Medical Record System Integrating Imaging, Cardiac Catheterization, and Surgical Records.

D: Longitudinal care by the same core team: long-term follow-up by a core physician team familiar with the patient's “complete medical history.”

E: A robust transition care system: a dedicated team ensures the seamless transfer of adolescents from pediatric to adult healthcare.

23. __ Training of CHD Specialists: In your opinion, what is the most pressing need for improvement in the current system?

A: Creation of a structured training and accreditation pathway for an independent subspecialty in CHD treatment

B: Enhance cross-training between interventional and surgical disciplines, for instance, by having interventionalists learn the surgical perspective and surgeons master basic catheter skills.

C: Establish comprehensive training programs that cover both pediatric and adult CHD, thereby bridging the patient-age divide.

D: Promote the inclusion of interventional cardiologists as core members of the surgical team during complex CHD procedures.

E: Increase access to simulation-based training targeting rare and high-risk procedures.
